# Reducing Loneliness and Social Isolation Through the HELPeN Telephone-Call Program: Results from a Randomized Controlled Trial in Older Adults Living in the Community

**DOI:** 10.3390/jcm15010093

**Published:** 2025-12-23

**Authors:** María Jesús Hernández-López, Jessica García-González, César Leal-Costa, Antonio Jesús Ramos-Morcillo, Isidora Díaz-García, María Verónica López-Pérez, Solanger Hernández-Méndez, María Ruzafa-Martínez

**Affiliations:** 1 Faculty of Social and Health Sciences, University of Murcia, 30800 Lorca, Spain; mariajesus.hernandez2@um.es (M.J.H.-L.); isidora.diaz@um.es (I.D.-G.); mariaveronica.lopez@um.es (M.V.L.-P.); solanger.hernandez@um.es (S.H.-M.); 2 Faculty of Health Sciences, University of Almeria, 04120 Almería, Spain; 3 Faculty of Nursing, University of Murcia, 30120 Murcia, Spain; ajramos@um.es (A.J.R.-M.); maruzafa@um.es (M.R.-M.)

**Keywords:** loneliness, social isolation, aged, social support, depression, cognitive dysfunction, randomized controlled trial

## Abstract

**Background/Objectives:** Loneliness is a significant public health issue among older adults, especially in rural and socioeconomically vulnerable groups. Telephone-based interventions have become a scalable, cost-effective way to reduce social isolation, although evidence of their long-term effects on various health outcomes remains limited. This study aimed to assess how effective HELPeN, a structured telephone program delivered by trained nursing students, is in decreasing loneliness and enhancing psychosocial and cognitive health in community-living older adults. **Methods:** A randomized controlled trial was conducted with 119 older adults (≥65 years) residing in the community. Participants were allocated to either an intervention group (n = 65), which received weekly structured telephone calls over 9 months, or a control group (n = 54), which received standard care. Outcomes were evaluated at baseline (M0), mid-intervention (M1–M3), and 3 months after the intervention (M4). The primary outcomes measured included loneliness and perceived social support. Secondary outcomes comprised functional status, comorbidities, depressive symptoms, quality of life, sleep quality, and cognitive function. The data were analyzed using repeated-measures ANOVA with Greenhouse–Geisser correction. **Results:** Significant group interactions over time were identified for loneliness (F = 5.92, *p* = 0.001, η^2^ = 0.067), social support (F = 3.39, *p* = 0.023, η^2^ = 0.043), depressive symptoms (F = 3.87, *p* = 0.019, η^2^ = 0.046), and cognitive status (F = 5.35, *p* = 0.002, η^2^ = 0.063). No significant differences were found for functional status, comorbidity, sleep quality, or quality of life. **Conclusions:** The HELPeN program demonstrated significant effectiveness in reducing loneliness and social isolation, and in improving emotional, cognitive, and sleep-related outcomes in older adults. As a low-cost and scalable model, this intervention strengthens the role of nursing students in addressing social determinants of health and may be integrated into community and public health strategies targeting vulnerable aging populations.

## 1. Introduction

Loneliness and social isolation have emerged as two of the primary public health challenges associated with the aging population. Although often used interchangeably, these terms represent distinct phenomena. Loneliness is defined as “an aversive state experienced when there is a discrepancy between desired interpersonal relationships and those perceived at any given moment” [1] and also as “a persistent state of emotional discomfort that arises when an individual feels rejected by others or lacks companions to engage in activities that foster social integration and emotional intimacy” [2]. Recently, it has been acknowledged as a social determinant of health [3]. Conversely, social isolation pertains to a more objective, structural dimension, relating to the quantity and frequency of genuine social interactions, and is characterized as “the objective absence or scarcity of contacts and interactions between an individual and a social network.” Both concepts are closely interconnected and tend to coexist in older adults [4].

The World Health Organization (WHO) estimates that loneliness affects one in six individuals globally and is associated with more than 871,000 deaths annually [3]. Social isolation also constitutes an escalating public health concern; a recent study using data from 159 countries worldwide found that its prevalence increased from 19.2% in 2009 to 21.8% in 2024 [5]. In Spain, it is reported that 20% of individuals over the age of 65 experience high levels of loneliness, while 25% suffer from social isolation [6]. The most affected demographics include women, the elderly, individuals with low educational and economic status [6], those with mobility limitations, disabilities [7], low digital literacy, and diminished social networks [8,9]. Additionally, factors such as living without a partner, having a limited social network, and infrequent interactions with grandchildren or neighbors are correlated with these issues [10].

Both loneliness and social isolation are predictors of diminished quality of life in adults aged 65 years or older [11], influenced by factors such as physical, emotional, and functional health, and social participation [12,13]. Evidence demonstrates that loneliness and social isolation adversely impact physical and mental health, elevating the risk of cognitive decline [14], depression, anxiety [15], sleep disorders [16], and mortality, with 14% of deaths attributable to loneliness [17]. Furthermore, the association between loneliness and social isolation and an increased risk of myocardial infarction and stroke has been documented, with magnitudes comparable to risk factors such as smoking, obesity, and physical inactivity [6,17]. Consequently, health services must address both loneliness and social isolation to enhance the well-being and health outcomes of this demographic group [11].

Community interventions and social support strategies are vital to preserving well-being among the elderly. Remote communication methods, such as telephone and video calls, have demonstrated efficacy in reducing perceptions of loneliness, regardless of the specific technology used, underscoring the utility of accessible, cost-effective approaches [18]. Telephone-based interventions are regarded as a practical and effective method to alleviate loneliness among older adults, particularly in contexts with limited mobility or in rural regions. Such interventions have the potential to enhance overall well-being and reduce loneliness, especially when personalized to individual needs and aimed at reinforcing social support and fostering positive cognitive processes [19,20].

Various international trials have demonstrated the effectiveness of structured telephone interventions in reducing loneliness and improving the health of older individuals. In the United Kingdom, the BASIL+ Trial demonstrated a 21% decrease in loneliness levels, along with improvements in depression and quality of life after 8 weeks of telephonic contact, with the benefits sustained post-intervention [21]. In the United States, a six-month telephony program succeeded in notably decreasing loneliness from 28% to 46%, anxiety from 23% to 63%, and days of poor physical and mental health [22]. The HEAL-HOA initiative involving telephone volunteering among the elderly revealed significant reductions in loneliness, stress, and depressive symptoms, and improvements in social networks [23]. Lastly, in Sweden, a telephone intervention implemented in primary care demonstrated improvements in functioning, quality of life, anxiety, and loneliness at 3 and 6 months [24].

Conversely, intergenerational programs that connect young volunteers or students of health sciences with older adults appear to diminish loneliness and foster a more positive perception of aging among the students themselves [25]. For instance, weekly 30 min calls have been held between university students and seniors over 8 weeks, enhancing emotional well-being; 80% of the elderly reported an improved sense of well-being following the conversations [26]. Similarly, another intervention involving weekly hour-long sessions facilitated by college students demonstrated significant improvements in loneliness, life satisfaction, and depressive symptoms among older participants [27]. Several randomized clinical trials have substantiated the effectiveness of telephone interventions in alleviating loneliness. Two recent clinical trials, following four weeks of calls, observed significant reductions in UCLA scores relative to control groups, with mean decreases ranging from 0.5 to 1.1 points, particularly when the calls incorporated empathy and emotional support elements [28,29]. Only one of these trials reported additional enhancements in other indicators such as depression, anxiety, and cognitive function [28].

Nevertheless, certain studies have not demonstrated positive effects in alleviating loneliness. For instance, the 21st Century Good Neighbor Program [30] and other qualitative studies [31,32] do not facilitate an objective quantification of the magnitude of such interventions. A recent systematic review analyzed 27 digital intergenerational programs aimed at reducing loneliness and social isolation among older adults, emphasizing that effectiveness depends on alignment among the employed strategy, the context, and the mechanisms activated. It underscores the significance of continuous support and tailoring interventions to the specific characteristics of the elderly [33].

To date, research remains limited, presenting methodological particularities that complicate the formulation of definitive conclusions. Pilot studies with small sample sizes have been identified [34], often lacking control groups [26,27], featuring brief follow-up periods and without post-intervention phases [29], and employing non-validated measurement scales or exclusively qualitative outcomes [31,32].

Interventions addressing loneliness and social isolation have varied widely in duration, training, and content, limiting their capacity to produce sustained effects. Programs such as Tellegacy [27], and the empathy-focused calls [29] were restricted to brief four-week feasibility phases, while most relied on minimally trained volunteers—typically younger intergenerational callers—who received only one to three hours of preparation [26,27,29]. Intervention content varied substantially across programs, with Tellegacy centering its approach on structured oral reminiscence [27], the Social Bridging Project [26], emphasizing technology-based engagement and digital skill development, and HEAL-HOA [23], implementing targeted psychosocial modalities delivered by trained older adult interventionists.

HELPeN was explicitly designed to address the limitations of prior interventions through a robust and coherent structure. It delivered weekly 30 min calls over 36 consecutive weeks, implemented by nursing students who completed an intensive 25 h training program including clinical simulation. Its structured social dialog script emphasized emotional validation and meaningful memories, ensuring both consistency and therapeutic depth. Conceptually, the intervention draws on the social-ecological model of loneliness and social isolation [35], which highlights how individual, interpersonal, community, and societal factors jointly influence older adults’ risk of loneliness and isolation. Additionally, HELPeN is informed by resilience-based frameworks for aging and social isolation, which highlight that enhancing older adults’ coping self-efficacy, integration of social support, and adaptive capacity can buffer the negative psychological and health impacts associated with social isolation and loneliness [36].

Consequently, the primary objective of this study was to assess the effectiveness of HELPeN, a weekly 30 min telephone intervention, administered over 36 weeks by nursing students trained through structured clinical simulation, to reduce loneliness and social isolation among older adults residing in the community. As secondary endpoints, its impact on depression, sleep quality, cognitive impairment, functionality, quality of life, and the Charlson comorbidity index was examined, with measurements taken at baseline, every three months, and three months post-intervention.

## 2. Materials and Methods

### 2.1. Design

A two-arm, parallel, randomized clinical trial was conducted, comprising an intervention group and a control group, with a duration of 12 months. Throughout the study, a baseline assessment (M0 = before intervention), three intermediate assessments (M1 = 3 months, M2 = 6 months, M3 = 9 months), and a final assessment (M4 = 3 months post-intervention) were conducted. This clinical trial is registered with ClinicalTrials.gov (U.S. National Library of Medicine (NLM) at the National Institutes of Health (NIH) (Bethesda, MD, USA) under the registration number NCT05644288 and registration date: 9 December 2022.

### 2.2. Sample

The study population consisted of individuals aged 65 or older residing in Health Area 3 of the Region of Murcia, which encompasses the municipalities of Lorca, Águilas, Puerto Lumbreras, and Totana. In total, this demographic comprises 29,955 persons aged 65 or older, distributed as follows: Lorca (15,148), Águilas (6905), Puerto Lumbreras (2741), and Totana and Aledo (5161) [37,38,39,40,41]. Within this particular health domain, the HELPeN project determined that 31.4% of elderly individuals experience unwanted loneliness, while 14.7% perceive themselves as socially isolated [42].

The inclusion criteria were being 65 years of age or older, residing in the community (not being institutionalized), and belonging to Health Area 3 of the Region of Murcia. In addition, participants were required (1) to present loneliness and/or low perceived social support (score < 32 on the Duke-UNC-11 Scale and/or ≤30 on the UCLA Loneliness Scale) and (2) to have no cognitive impairment (defined as ≤2 errors on the Pfeiffer Questionnaire). Participants who had moderate or severe cognitive impairment (≥3 errors on the Pfeiffer Questionnaire), hearing or language difficulties that impeded telephone communication, or who participated in other social or psychological support programs were excluded. The causes of study termination were voluntary abandonment of the patient, non-compliance with the protocol (they did not respond to the volunteer’s call), and death.

To ensure the sample’s representativeness, the sample size was calculated using Pass 11 (Power Analysis & Sample Size) (NCSS, Kaysville, UT, USA) software for the ANOVA repeated measures test [43]. Each subject within each group undergoes five measurements. Considering an alpha level of 0.05, an expected effect size of 1.58, a beta power of 1, and an N2/N1 allocation ratio of 1, the minimum required sample size per group is 35, resulting in a total sample size of 70 subjects. To account for potential losses during follow-up, this number is increased by 20%, resulting in a requirement of 42 subjects per group.

### 2.3. Procedure and Randomization

Participants were recruited through professionals at all primary care health centers within Health Area III. The nurses were briefed on the study’s objectives and inclusion criteria, enabling them to identify and recruit eligible elderly individuals. Following the informed consent process, the nurses provided a list of interested contacts. Additionally, social workers from the health area collaborated in the process, and informational posters were displayed to allow interested elderly individuals or their relatives to contact the research team directly. Subsequently, a member of the research team contacted potential participants by telephone, assessed their eligibility according to the inclusion criteria, and invited them to participate.

Participants who provided their informed consent were registered on the REDHI^®^ digital platform, specifically developed for this study. The computer application utilized an algorithm for the random allocation of subjects to the study groups, either the control group (CG) or the experimental group (EG), ensuring the impartiality of the process and preventing any influence from the research team.

Participants assigned to the CG were informed that they would receive a follow-up call in three months and were provided with the name of their evaluator. Participants in the EG were similarly informed of their evaluator’s name and additionally notified that they would be contacted in three months for follow-up assessments; they were also provided with the name of the volunteer responsible for conducting the calls.

### 2.4. Intervention: HELPeN Program

The intervention group participated in the HELPeN program, a structured telephone intervention developed by nursing students from the University of Murcia, who had previously undergone training and supervision by research nurses. The students completed the HELPeN training program, which entailed a total of 25 h of instruction distributed over three weeks. This training comprised an asynchronous theoretical component, conducted online via a virtual platform, consisting of five modules to be completed sequentially before progressing to the next phase. The subsequent phase involved a synchronous practical component, featuring a five-hour online session conducted via a videoconferencing platform, which simulated telephone call scenarios. Following the completion of the program, notable improvements were documented in students’ attitudes towards the elderly, with increased positivity post-training and enhanced knowledge of interventions to address loneliness and isolation among the elderly [44].

During the academic years 2022–2023 and 2024–2025, a total of 109 students enrolled in the second and third years of the bachelor’s degree in nursing at the University of Murcia, comprising 13 males and 96 females. Out of these, 55 students (7 males and 48 females) completed the HELPeN training program. In total, 38 volunteer participants (4 males and 34 females) were registered, representing 31 individuals, as six females and one male (a total of 7 volunteers) participated in more than one academic year. Each volunteer was limited to making calls to a maximum of two elderly people per academic year.

The intervention consisted of weekly calls lasting approximately 30 min, conducted over 36 consecutive weeks. These calls concentrated on social dialog, attentive listening, emotional validation, and the encouragement of meaningful memories, interests, activities, and a focus on change. Each session adhered to a structured script and was documented on the REDHI^®^ platform (Universidad de Murcia, Murcia, Spain), ensuring procedural fidelity.

Volunteers had access to the digital platform, where they could consult the sociodemographic information of the elderly assigned to them and record data for each call. The registration module included the start and end date and time of each call, to calculate its duration, the schedule of the next session, the number of calls, and aspects related to the development of the intervention, such as the need to redirect the conversation, the achievement of the planned objectives, the satisfaction of the volunteer, and the main objective of the session. Additionally, they had a free text field to note relevant observations or incidents.

They also had a dedicated nursing section that integrated records of assessment, diagnosis, and interventions, in accordance with the nursing process methodology.

The control group did not receive supplementary calls; their participation was confined to telephone interviews necessary for data collection at various measurement points in time.

### 2.5. Variables and Measuring Instruments

The variables were assessed at five distinct time points: a baseline measurement before the commencement of the intervention (M0), at three months (M1), at six months (M2), at nine months (M3, corresponding with the conclusion of the intervention), and at twelve months from the initiation (M4, three months after the conclusion of the intervention).

Primary outcomes 

The primary variables examined were loneliness and social isolation. Loneliness was assessed using the UCLA Loneliness Scale, comprising 10 items rated on a Likert scale from 1 to 4, yielding a total score ranging from 10 to 40. A score below 20 indicates severe loneliness, while a score between 20 and 30 indicates moderate loneliness [45]. Social isolation or perceived social support was evaluated using the Duke-UNC-11 Scale, which ranges from 11 to 55 points; scores below 32 indicate low social support [46].

Secondary outcomes 

As a secondary variable, depression was evaluated utilizing the abbreviated Yesavage Depression Scale (GDS), consisting of 15 items. The score ranges from 0 to 15 points; scores between 5 and 8 indicate mild depressive symptoms, between 9 and 11 moderate symptoms, and scores greater than 12 indicate severe symptoms [47].

The quality of life was evaluated by employing the EuroQol-5D-5L (EQ-5D) instrument. It characterizes the health status across five dimensions—mobility, personal care, daily activities, pain/discomfort, and anxiety/depression—each comprising five levels of severity, ranging from 1 (no problems) to 5 (extreme problems). The combination of these five values produces a code that signifies the participant’s health condition. This code is subsequently converted into a Utility Index, which ranges from 1 (indicating perfect health) to negative values (indicating conditions worse than death), extending down to 0 (representing a state akin to death) [48].

Comorbidity was assessed using the age-adjusted Charlson Index, wherein higher scores indicate a greater burden of disease [49]. Sleep quality was evaluated using the Pittsburgh Sleep Quality Index (PSQI), which ranges from 0 to 21 points; scores exceeding 5 indicate poor sleep quality [50].

Cognitive impairment was assessed using the 10-item Pfeiffer Questionnaire (SPMSQ), with scores of 2 or fewer errors indicating normal cognitive functioning and scores of 3 or more indicating cognitive impairment [51]. Finally, functional status was evaluated using the Barthel Index, which ranges from 0 to 100, with higher scores indicating greater independence in essential activities of daily living [52].

Furthermore, data were collected on demographic attributes, including age, gender, marital status, educational level, number of children, income, rural or urban setting, and cohabitation.

### 2.6. Data Collection and Processing

All sources of information were primary, obtained directly from the participants through structured telephone interviews conducted by the blinded researchers, at the five time points (M0–M4).

The data were recorded directly on the REDHI^®^ platform (Universidad de Murcia, Murcia, Spain), previously validated through pilot tests that guaranteed its reliability for data recording and storage, and designed specifically for this project. Personal data were separated from clinical data and encrypted; only the principal investigators had access to the complete database, thereby ensuring confidentiality and compliance with the General Data Protection Regulation (EU 2016/679).

### 2.7. Ethical Considerations

The research received approval from the Ethics Committee of the University of Murcia (No. 3267/2021) and the Management of Health Area III of the Region of Murcia. All participants were duly informed of the study’s objectives and provided written informed consent before participating. Furthermore, participants were advised of their right to withdraw from the study at any point. The study was conducted in accordance with the principles outlined in the Declaration of Helsinki and complied with the European Data Protection Regulation (EU 2016/679).

### 2.8. Data Analysis

A descriptive univariate analysis of the independent and dependent variables collected throughout the study was conducted, using measures of central tendency (mean and standard deviation) and examining distribution types to determine their fit to normality. For the qualitative variables, both absolute and relative values were calculated. A bivariate analysis was performed to assess the homogeneity of the experimental and control groups concerning the variables studied. In this context, the Chi-Square test and Student’s *t*-test were employed, depending on the nature of the comparison variable.

To assess the impact of the intervention on the dependent variables, an intention-to-treat (ITT) analysis was conducted, comparing outcomes for social isolation, loneliness, depression, cognitive impairment, functional status, sleep quality, comorbidity, and quality of life. A one-factor repeated measures ANOVA was employed to analyze intra-subject differences, while a two-factor repeated measures ANOVA was utilized to examine inter-subject differences.

All results will be regarded as statistically significant when the *p*-value is less than 0.05. Data analysis will be conducted using SPSS^®^ v. 26 (Statistical Package for the Social Sciences) (IBM Corporation, Armonk, NY, USA).

## 3. Results

### 3.1. Sample Description

**Figure 1:**
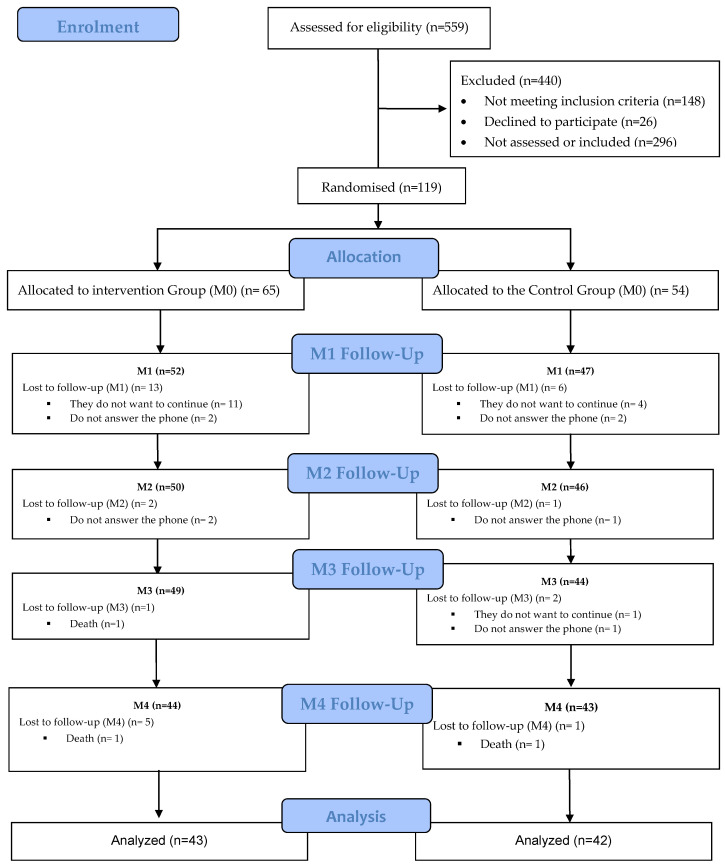
Consort Flow Diagram.

The initial sample comprised 119 participants, with 54 allocated to the CG and 65 to the EG. However, due to attrition, the final sample consisted of 42 individuals in the experimental group and 43 in the control group (Figure 1). As delineated in Table 1, no statistically significant differences were observed between the two groups concerning the sociodemographic variables examined.

The average age of the participants was 76.36 years (SD = 6.47), with no statistically significant differences observed between the control group (75.43 years) and the experimental group (77.13 years). Concerning gender distribution, 74.8% of the entire sample were female, with comparable proportions in both groups.

Concerning educational attainment, most participants did not possess formal education (56.3%), followed by those with primary education (37%). A minority had completed secondary education (0.8%) or university studies (0.8%), with no statistically significant differences observed between the groups.

Regarding marital status, 52.1% of participants were widowed, while 26.9% were married and 9.2% were single. The distribution was comparable across the CG and the EG. Similarly, most participants resided in urban areas (77.3%). However, a slightly higher proportion of rural residents was observed in the EG (29.2%) compared to the CG (14.8%), a difference that was not statistically significant.

In terms of economic income, most participants (85.8%) reported earning between €500 and €999 per month. Only one participant reported an income below €500, and 13.4% reported earnings between €1000 and €1499, with no significant differences between the groups.

Regarding the number of children, the majority had 2 (35.4%) or 3 (26.9%). The remaining categories showed similar distributions across groups, with no significant differences. Additionally, regarding cohabitation, 52.1% of participants lived alone, 39.5% lived with one other person, and smaller proportions lived with two or three people. No significant differences were observed between the CG and EG for this variable.

Overall, both groups exhibited comparable sociodemographic characteristics at the outset of the study, thereby ensuring the necessary homogeneity for the subsequent analysis of the intervention’s effects.

### 3.2. Effect of the Intervention

**Figure 2:**
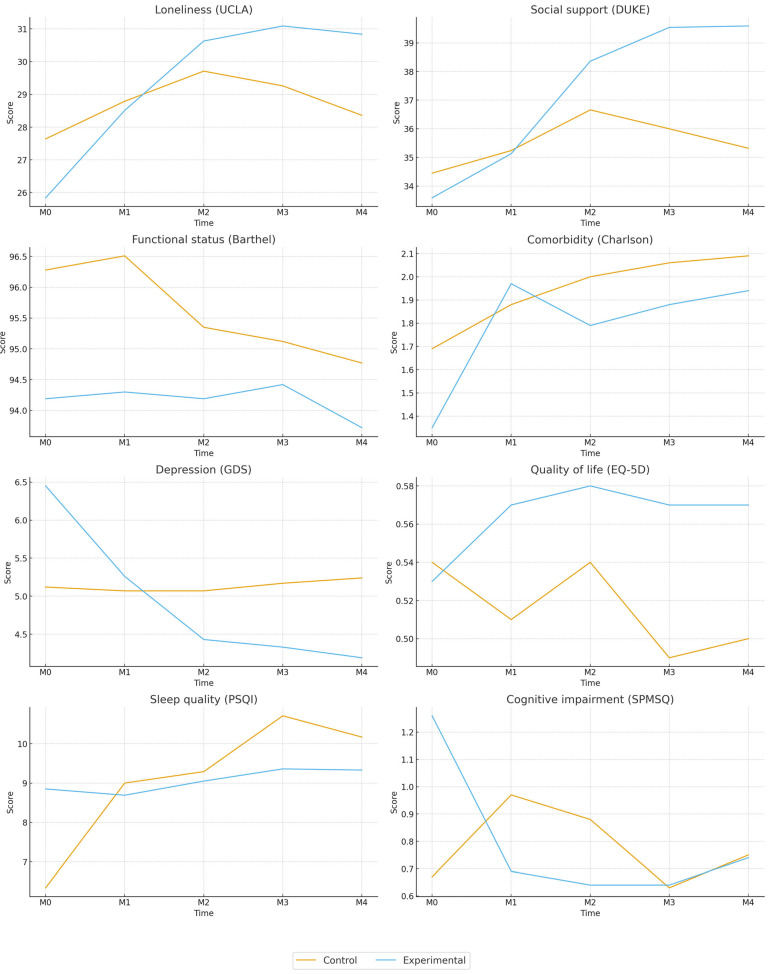
Line graphs showing changes in all measurements over time for both the experimental and control groups in the variables.

A repeated measures analysis was performed with an intra-subject factor of Time (M0–M4) and an inter-subject factor of group (experimental versus control). Due to the significant result of Mauchly’s sphericity test (*p* < 0.001), the Greenhouse–Geisser correction was applied to all contrasts. The results are shown in Table 2 and Figure 2.

Regarding the loneliness variable (UCLA), the intra-subject analysis revealed a significant effect of time in the experimental group [F = 12.07 (4, 80), *p* < 0.001, η^2^ = 0.376], with a 5-point increase corresponding to approximately 16.6% of the scale range, indicating a substantial change in perceived loneliness. The control group also exhibited a smaller yet statistically significant effect [F = 3.09 (4, 80), *p* = 0.02, η^2^ = 0.13], with an intermediate effect size. A significant interaction between time and group [F = 5.92 (2.90, 240.97), *p* < 0.001, η^2^ = 0.067] was observed, with a medium effect size, suggesting that the experimental group improved over time. In contrast, the control group demonstrated a more moderate pattern that eventually declined.

For the perceived social support (DUKE) variable, a significant main effect was observed in the experimental group [F = 7.94 (4, 80), *p* < 0.001, η^2^ = 0.31], with a 6-point increase corresponding to approximately 13.6% of the scale range, indicating a substantial improvement in perceived social support. Conversely, the control group did not demonstrate significant changes over time. The interaction between time and group was also significant [F = 3.39 (2.67, 199.96), *p* = 0.023, η^2^ = 0.043], with a small effect size, reflecting a progressive increase exclusive to the experimental group.

Regarding the variable of functional status (Barthel Index), no significant main effects were identified in the experimental group [F = 0.72 (4, 80), *p* = 0.58, η^2^ = 0.034] nor in the control group [F = 1.73 (4, 80), *p* = 0.15, η^2^ = 0.079]. Furthermore, the time*group interaction was not significant [F = 0.610 (2.08, 174.68), *p* = 0.551, η^2^ = 0.007], indicating stability in functional measures across both groups.

For the comorbidity variable (Charlson Index), there was no statistically significant main effect of time observed within the experimental group [F = 1.736 (4, 80), *p* = 0.15, η^2^ = 0.102], nor within the control group [F = 0.795 (4, 80), *p* = 0.53, η^2^ = 0.050]. Additionally, the interaction between time*group was not significant [F = 0.559 (1.62, 103.58), *p* = 0.54, η^2^ = 0.009], indicating stability in the measurements across both groups.

In the depression variable (GDS), the experimental group demonstrated a statistically significant main effect [F = 3.154 (4, 80), *p* = 0.019, η^2^ = 0.14], with a 2.26-point decrease corresponding to approximately 15.1% of the scale range, indicating a meaningful reduction in depressive symptoms. Conversely, the control group exhibited no significant changes over the evaluated period. A noteworthy interaction between time and group [F = 3.876 (2.19, 177.68), *p* = 0.019, η^2^ = 0.046] was identified, with a modest effect size, suggesting that the progression of depressive symptoms varied between groups, with a decline observed in the experimental group.

Within the parameter of quality of life (EQ-5D), no significant effects of time were observed in the experimental group [F = 0.496 (4, 80), *p* = 0.74, η^2^ = 0.028], nor in the control group [F = 1.891 (4, 80), *p* = 0.122, η^2^ = 0.100]. Furthermore, the interaction between time and group was not statistically significant [F = 1.212 (2.75, 195.12), *p* = 0.306, η^2^ = 0.017], indicating no evident differences in the temporal trajectory between the groups.

For the sleep quality (PSQI) variable, the experimental group did not show significant changes [F = 0.352 (4, 80), *p* = 0.842, η^2^ = 0.018]. Conversely, the control group demonstrated a significant main effect [F = 13.724 (4, 80), *p* < 0.001, η^2^ = 0.423], with a large effect size, indicating a progressive increase in the score, suggesting worsening of sleep quality. A statistically significant interaction between time and group was observed [F = 6.262 (3.17, 247.52), *p* < 0.001, η^2^ = 0.074], with a moderate effect size, suggesting that the temporal progression of sleep quality differed between the two groups. Notably, the experimental group maintained a more stable trajectory, whereas the control group showed a progressive increase in score, reflecting a decline in sleep quality.

Finally, regarding the cognitive impairment variable (SPMSQ), the experimental group showed a statistically significant main effect of time [F = 3.964 (4, 80), *p* = 0.006, η^2^ = 0.17], with a 0.52-error decrease corresponding to approximately 5.2% of the scale range, indicating a modest improvement in cognitive function. No statistically significant alterations were observed within the control group. The interaction effect between time and group was also significant [F = 5.351 (2.73, 218.21), *p* = 0.002, η^2^ = 0.063], with a medium effect size, suggesting a more pronounced pattern of improvement in the experimental group.

## 4. Discussion

The present study evaluated the effectiveness of the HELPeN program, a structured telephone intervention conducted by nursing students in reducing loneliness and social isolation among older individuals residing in the community. The results demonstrated significant increases in perceived social support and significant decreases in loneliness, depressive symptoms, and cognitive impairment in the experimental group, with moderate-to-large effect sizes. Conversely, no significant changes were observed in comorbidity, functioning, sleep quality, or quality of life. These findings substantiate that telephone interventions, grounded in emotionally meaningful social companionship, can produce sustained and multidimensional health benefits for older adults.

Our sample was composed of older adults experiencing loneliness and social isolation, with a predominance of women and a substantial proportion of individuals with low educational attainment and limited economic resources, indicating a profile of social vulnerability. This pattern closely aligns with demographic data for older adults in Spain: according to the Spanish Institute of Statistics, only 16–17% of people aged 65 and over have higher education, while more than 70% remain at basic educational levels [53]. A similar distribution was observed in our previous study conducted in the same health area, where 52.9% were women and 69.6% had no formal or only primary education [40]. In addition, the majority of participants (85%) reported a monthly income between €500 and €999. In Spain, the average pension is €1314 per month, and the average survivor’s pension is €936.3, the main income for 1.5 million people, 95.7% of whom are women [54]. This socioeconomic pattern is further exacerbated among older women, who in Spain experience significantly lower income and higher economic vulnerability due to lifelong gender disparities in employment and pension accumulation [55]. Evidence has also demonstrated that lower education and lower income increase vulnerability to loneliness and social isolation in older adults, partly due to reduced social resources and fewer opportunities for participation [56].

Previous trials using telephonic or volunteer-based interventions have shown modest to moderate reductions in loneliness, although with significant variations in their intensity, duration, and therapeutic approach. This evidence, which includes brief empathy-focused calling protocols [29], structured wisdom-based narrative therapy [28], and multi-session behavioral activation programs [21], supports the idea that empathetic and regular telephone communication is an effective and accessible way to reduce loneliness. A meta-analysis by Fu et al. further strengthens this conclusion [20]. Building on this foundation, our study implemented a longer and more intensive intervention—a nine-month program of weekly 30 min calls delivered by nursing students, with a follow-up assessment three months after the intervention. This sustained, high-dose model likely explains the notable improvement in loneliness observed (5 points; 16.6%), suggesting that longer-term programs facilitated by trained volunteers from health-related fields may produce more significant and lasting effects than shorter or less intensive alternatives.

Our study demonstrated that the intervention produced a substantial increase in perceived social support, with participants’ scores on the Duke-UNC-11 Scale rising from 33 at baseline to 39.6 at 12 months, remaining well above the threshold for low social support (<32) for up to 3 months post-intervention. This improvement is likely due to the 9-month, telephone-based interactions with nursing students, which provided consistent, proactive companionship that combined health monitoring, reminiscence, and goal setting, serving as a stable relational anchor. In contrast, the BASIL+ trial [21] found behavioral activation ineffective in reducing measurable social isolation, whereas HEAL-HOA [23] showed that structured volunteering could successfully expand objective social networks. These findings suggest that social health interventions can operate through different pathways: some enhance structural connections, whereas others, like ours, primarily strengthen the subjective experience of support and well-being.

The intervention yielded notable mental health benefits, with reductions in depressive symptoms and improvements in cognitive performance [27,29]. Reminiscence, implemented in the HELPeN program, may have contributed to alleviating depression by facilitating emotional processing, recalling positive memories, strengthening identity, and promoting social interaction [57], while potentially supporting cognitive function through engagement of neural networks involved in autobiographical memory and executive function [58,59]. Additionally, the stabilization of sleep quality in the experimental group, compared with deterioration in the control group, suggests that sustained social interactions provide holistic benefits for psychological, cognitive, and physiological well-being [14,16].

Conversely, the lack of changes in functionality (Barthel), quality of life (EQ-5D), and comorbidity (Charlson) may be interpreted as indicative of positive stability in variables that generally tend to decline with age. Nevertheless, these findings also highlight potential limitations of the intervention type, which focuses on emotional and social aspects rather than functional or physical ones. Integrating social interventions with physical or multimodal components could exert a greater influence on overall health.

From a practical standpoint, this study has significant implications for public health policies and nursing practice. Using nursing students is not only innovative but also demonstrates a sustainable and replicable model that offers dual benefits: it enhances the emotional well-being of the elderly and raises awareness while training future health professionals in biopsychosocial care. Moreover, it is a low-cost, scalable, and adaptable intervention suitable for various settings, making it a valuable resource for health systems with limited resources or dispersed populations, such as the rural areas examined in this study.

Several therapeutic mechanisms might explain the success seen in the intervention group. Regular, structured phone calls likely fostered a sense of continuity, emotional safety, and support availability—factors known to reduce feelings of loneliness and social disconnection [28,29]. The call structure, focused on active listening, validation, and encouragement, probably boosted perceived social support by targeting cognitive and emotional aspects of isolation. Additionally, empathetic engagement by nursing students may have served as a meaningful social stimulus, increasing motivation, self-efficacy, and adaptive coping skills, which are linked to depressive symptoms and perceived health. The intervention may also have enhanced social-cognitive aspects such as trust, reciprocity, and positive expectations about social contact, which could indirectly improve mood, sleep, and cognitive performance [21,22,23]. Overall, these therapeutic factors offer a plausible explanation for how a low-intensity, phone-based social support program can lead to measurable improvements in psychosocial and cognitive outcomes in older adults.

Nevertheless, the current study possesses several limitations. Firstly, despite an adequate sample size and randomization, the investigation was confined to a single region within the country, thereby restricting the generalizability of the findings. Secondly, participant attrition, although anticipated in longitudinal studies involving older populations, may introduce biases, particularly in self-reported assessments. Additionally, the specific socioeconomic profile, characterized by lower education and financial resources, may limit the generalizability of the results. Future research should include more socioeconomically diverse participants to better capture potential variations across groups. Furthermore, although validated and reproducible scales were employed, data collection via telephone could potentially engender social desirability biases. Lastly, the intervention lacked a qualitative evaluation of subjective experiences, an element that could augment the comprehension of the therapeutic process and its personal significance.

Based on these results, future research should explore hybrid interventions that combine telephone contact with face-to-face or digital group activities, as well as multicomponent interventions that include physical activity, cognitive stimulation, or community support. It would be pertinent to investigate the impact on vulnerable subgroups (e.g., individuals over 80 years of age, persons with disabilities, or residents of rural areas). Additionally, it would be beneficial to analyze the role of the quality of the volunteer-participant relationship through mixed-method approaches, including content analysis of calls or post-intervention interviews. In this context, recent publications

Taken together, the results of this study offer compelling evidence regarding the effectiveness of structured telephone interventions in mitigating loneliness, enhancing social support, and improving the emotional and cognitive well-being of older adults. These findings contribute to the expanding body of international literature that regards social accompaniment interventions as a vital strategy to address the emerging challenge of loneliness amid aging populations.

## 5. Conclusions

The HELPeN structured telephone intervention, developed and implemented by nursing students, proved effective in mitigating unwanted loneliness, social isolation, and enhancing emotional, social, and cognitive parameters among elderly individuals residing within the community. The experimental group demonstrated significant improvements in loneliness, social support, depressive symptoms, and cognitive functioning, with moderate-to-large effect sizes, whereas the control group showed no beneficial changes and, in some cases, deterioration.

These findings substantiate that social interventions grounded in emotional support, sustained over a period and structured professionally, have the potential to engender psychological and functional benefits that surpass those achieved through mere telephone contact. Owing to its cost-effective design, scalability, and applicability in rural or resource-constrained settings, HELPeN emerges as a promising strategy to mitigate loneliness and social isolation among the elderly and to reinforce the role of community nursing.

## Figures and Tables

**Table 1:** Characteristics of the participants.

		Total (N = 122)	Control Group (N = 54)	Experimental Group (N = 65)	*p* Value
Age Mean (Standard Deviation)		76.36 (6.47)	75.43 (6.20)	77.13 (6.64)	0.152
Gender % (N)	Total	100.0 (119)	100.0 (54)	100.0 (65)	
	Female	74.8 (89)	75.9 (41)	73.8 (48)	0.795
	Male	25.2 (30)	24.1 (13)	26.2 (17)	
Educational level % (N)	Total	100.0 (119)	100.0 (54)	100.0 (65)	
	No Studies	56.3 (67)	57.4 (31)	55.4 (36)	0.722
	Primary Education	37.0 (44)	35.2 (19)	38.5 (25)	
	Secondary education	5.9 (7)	7.4 (4)	4.6 (3)	
	University Studies	0.8 (1)	0.0 (0)	1.5 (1)	
Marital status % (N)	Total	100.0 (119)	100.0 (54)	100.0 (65)	
	Single	9.2 (11)	7.7% (5)	9.2 (6)	0.909
	Married	26.9 (32)	24.6% (16)	24.6 (16)	
	Divorced	4.2 (5)	3.1% (2)	4.6 (3)	
	Separated	7.6 (9)	7.7% (5)	6.2 (4)	
	Widow or widower	52.1 (62)	40.0% (26)	54.5 (36)	
Setting % (N)	Total	100.0 (119)	100.0 (54)	100.0 (65)	
	Rural	22.7 (27)	14.8 (8)	29.2 (19)	0.115
	Urban > 50,000	23.5 (28)	22.2 (12)	24.6 (16)	
	Urban < 50,000	53.8 (64)	63 (34)	46.2 (30)	
Income % (N)	Total	100.0 (119)	100.0 (54)	100.0 (65)	
	Less than €500	0.8 (1)	0.0 (0)	1.5 (1)	0.436
	Between €500 and €999	85.8 (102)	83.3 (45)	87.7 (57)	
	Between €1000 and €1499	13.4 (16)	16.7 (9)	10.8 (7)	
Number of children % (N)	Total	100.0 (119)	100.0 (54)	100.0 (65)	
	0	13.4 (16)	13 (7)	13.8 (9)	0.394
	1	9.2 (11)	3.7 (2)	13.8 (9)	
	2	35.4 (42)	37 (20)	33.9 (22)	
	3	26.9 (32)	25.8 (14)	27.8 (18)	
	4	10.1 (12)	11.1 (6)	9.2 (6)	
	5	3.4 (4)	5.6 (3)	1.5 (1)	
	6	0.8 (1)	1.9 (1)	0.0 (0)	
	10	0.8 (1)	1.9 (1)	0.0 (0)	
Cohabitants % (N)	Total	100.0 (119)	100.0 (54)	100.0 (65)	
	0	52.1 (62)	46.3 (25)	56.9 (37)	0.150
	1	39.5 (47)	40.7 (22)	38.5 (25)	
	2	7.6 (9)	13 (7)	3.1 (2)	
	3	0.8 (1)	0.0 (0)	1.5 (1)	

**Table 2:** Comparison of the variables scores across the five measures between the control and experimental groups.

												Within-Subject			Between Measure*Group	
	M0		M1		M2		M3		M4							
	*M*	*SD*	*M*	*SD*	*M*	*SD*	*M*	*SD*	*M*	*SD*	*F*	*p*	*η* ^2^	*F*	*p*	*η* ^2^
Loneliness (UCLA)																
Control	27.64	0.67	28.79	0.96	29.71	0.86	29.26	0.78	28.36	0.80	3.09	0.02	0.13	5.92	<0.001	0.067
Experimental	25.84 ^b,c,d,e^	0.67	28.51 ^a,c,d,e^	0.95	30.63 ^a,b^	0.85	31.09 ^a,b^	0.77	30.84 ^a,b^	0.79	12.07	<0.001	0.376			
Social support (DUKE)																
Control	34.45	1.36	35.24	1.76	36.66	1.53	36.00	1.46	35.32	1.45	1.20	0.32	0.06	3.39	0.023	0.043
Experimental	33.59 ^c,d,e^	1.34	35.13 ^c,d,e^	1.74	38.36 ^a,b^	1.51	39.54 ^a,b^	1.44	39.59 ^a,b^	1.43	7.94	<0.001	0.31			
Functional status (Barthel)																
Control	96.28	5.45	96.51	5.08	95.35	5.48	95.12	5.63	94.77	6.09	1.73	0.15	0.079	0.610	0.55	0.007
Experimental	94.19	8.20	94.30	7.74	94.19	8.66	94.42	7.80	93.72	9.43	0.72	0.58	0.034			
Comorbidity (Charlson)																
Control	1.69	1.31	1.88	1.07	2.00	1.14	2.06	1.05	2.09	1.17	0.795	0.53	0.050	0.559	0.54	0.009
Experimental	1.35	1.91	1.97	1.34	1.79	1.18	1.88	1.12	1.94	1.18	1.736	0.15	0.102			
Depression (GDS)																
Control	5.12	3.18	5.07	3.37	5.07	2.96	5.17	2.77	5.24	2.97	0.084	0.987	0.004	3.876	0.019	0.046
Experimental	6.45 ^c,d,e^	3.82	5.26 ^a^	3.57	4.43 ^a^	3.13	4.33 ^a^	2.91	4.19 ^a^	3.01	3.154	0.019	0.14			
Quality of life (EQ-5D)																
Control	0.54	0.18	0.51	0.19	0.54	0.19	0.49	0.18	0.50	0.19	1.891	0.122	0.100	1.212	0.306	0.017
Experimental	0.53	0.22	0.57	0.22	0.58	0.22	0.57	0.22	0.57	0.21	0.496	0.74	0.028			
Sleep quality (PSQI)																
Control	6.34 ^b,c,d,e^	3.67	9.00 ^a^	4.04	9.29 ^a,d^	3.77	10.71 ^a,c^	3.87	10.17 ^a^	3.91	13.724	<0.001	0.423	6.262	<0.001	0.074
Experimental	8.85	3.68	8.69	3.76	9.05	3.30	9.36	3.11	9.33	3.23	0.352	0.842	0.018			
Cognitive impairment (SPMSQ)																
Control	0.67	0.80	0.97	0.97	0.88	1.04	0.63	0.84	0.75	1.08	2.424	0.055	0.112	5.351	0.002	0.063
Experimental	1.26 ^b,c,d^	1.08	0.69 ^a^	0.81	0.64 ^a^	1.03	0.64 ^a^	0.96	0.74	1.06	3.964	0.006	0.17			

M = Measure; M = Mean; SD = Standard Deviation; η^2^ = eta square. ^a, b, c, d, e^ indicates the measurement showing statistically significant differences with *p* < 0.05 in pairwise intragroup analysis.

## Data Availability

The data presented in this study are available on request from the corresponding author due to ethical reasons.
